# The impact of obstructive sleep apnea variability measured in-lab versus in-home on sample size calculations

**DOI:** 10.1186/1755-7682-2-2

**Published:** 2009-01-02

**Authors:** Daniel Levendowski, David Steward, B Tucker Woodson, Richard Olmstead, Djordje Popovic, Philip Westbrook

**Affiliations:** 1 Advanced Brain Monitoring Inc., Carlsbad, CA 92008, USA; 2 University of Cincinnati College of Medicine, Cincinnati, OH, USA; 3 Medical College of Wisconsin, Milwaukee, WI, USA; 4 University of California, Los Angeles, Los Angeles, CA, USA; 5 University of Southern California, Alfred Mann School of Biomedical Engineering, Los Angeles, CA, USA

## Abstract

**Background:**

When conducting a treatment intervention, it is assumed that variability associated with measurement of the disease can be controlled sufficiently to reasonably assess the outcome. In this study we investigate the variability of Apnea-Hypopnea Index obtained by polysomnography and by in-home portable recording in untreated mild to moderate obstructive sleep apnea (OSA) patients at a four- to six-month interval.

**Methods:**

Thirty-seven adult patients serving as placebo controls underwent a baseline polysomnography and in-home sleep study followed by a second set of studies under the same conditions. The polysomnography studies were acquired and scored at three independent American Academy of Sleep Medicine accredited sleep laboratories. The in-home studies were acquired by the patient and scored using validated auto-scoring algorithms. The initial in-home study was conducted on average two months prior to the first polysomnography, the follow-up polysomnography and in-home studies were conducted approximately five to six months after the initial polysomnography.

**Results:**

When comparing the test-retest Apnea-hypopnea Index (AHI) and apnea index (AI), the in-home results were more highly correlated (r = 0.65 and 0.68) than the comparable PSG results (r = 0.56 and 0.58). The in-home results provided approximately 50% less test-retest variability than the comparable polysomnography AHI and AI values. Both the overall polysomnography AHI and AI showed a substantial bias toward increased severity upon retest (8 and 6 events/hr respectively) while the in-home bias was essentially zero. The in-home percentage of time supine showed a better correlation compared to polysomnography (r = 0.72 vs. 0.43). Patients biased toward more time supine during the initial polysomnography; no trends in time supine for in-home studies were noted.

**Conclusion:**

Night-to-night variability in sleep-disordered breathing can be a confounding factor in assessing treatment outcomes. The sample size of this study was small given the night-to-night variability in OSA and limited understanding of polysomnography reliability. We found that in-home studies provided a repeated measure of sleep disordered breathing less variable then polysomnography. Investigators using polysomnography to assess treatment outcomes should factor in the increased variability and bias toward increased AHI values upon retest to ensure the study is adequately powered.

## Background

Obstructive Sleep Apnea (OSA) has recently gained recognition as one of the most common, under-diagnosed chronic diseases [1,2]. It is characterized by frequent loud snoring and recurrent failures to breathe adequately during sleep (termed apneas or hypopneas), as a result of full or partial collapse of the upper airway. OSA can be a confounding factor in clinical trials because it causes daytime drowsiness, and has been associated with hypertension, increased risk of congestive heart failure, coronary artery disease, myocardial infarction, cardiac arrhythmias, diabetes and stroke [3-8].

The most commonly referred prevalence statistic on obstructive sleep apnea syndrome is that 4% of men and 2% of women have the disease [9]. What doesn't get the same recognition is that 24% of the men and 9% of the women in the cohort were found to have sleep disordered breathing/obstructive sleep apnea (OSA)(i.e., AHI ≥ 5 without daytime somnolence). Because daytime somnolence and OSA severity are not well correlated [9,10] and there are individual differences in susceptibility to sleep deprivation [11,12], reliance on the former prevalence statistic could substantially underestimates the degree that this disease might affect subjects in clinical studies. More recent reports suggest the prevalence of OSA is increasing in part due to the rise in obesity, a major risk factor for OSA. In St. Louis MO, where the obesity rate is 28%, 19% of an adult surgical population was estimated to have moderate, severe or very severe OSA [13]. In a recent study of 331 dental patients, 59% of men and 22% of women were estimated to have an AHI > 10, and 43% and 12% were estimated to have an AHI > 20 [14].

In the United States laboratory polysomnography serves as the gold-standard for evaluating OSA. The PSG is used to obtain an overall AHI, and this is compared to some threshold value both for diagnosis and for treatment recommendations. In treatment outcome studies, however, the percentage change in AHI is the primary determinant of success. Thus, the inherent variability associated with the subject's, conditions, and the measurement tool become important factors, and it is presumed that the variability can be accurately estimated for purposes of sample size calculations.

The literature on repeated-measures PSG on symptomatic patients is somewhat limited. Chediak et al. [15] reported that 12 of 37 (32%) of their cases exhibited a difference in AHI ≥ 10 in two sequential nights of PSG. Le Bon et al. [16] studied 243 subjects during sequential nights of PSG and evaluated the benefit of improved sensitivity and specificity as a result of having multiple nights of data. Dean and Chaudhary [17] investigated PSG variability in nine patients who appeared negative during the first PSG study and positive in the second PSG. Carlile and Carlile [18] found that 48% of patients with an AHI < 5 had AHI values ≥ 5 on a repeat study.

Most recently Levendowski et al. [19] reported a fairly weak correlation (r = 0.44) between overall Apnea/Hypopnea indices from the two PSG studies conducted approximately 40 days apart with a 7 event/hour bias toward increasing AHI values upon retest.

The goal of this study was to assess the test – retest variability in untreated mild to moderate OSA patients studied with both a laboratory PSG and an in-home recording with a substantial time between the test and retest.

## Methods

The data were obtained from a multi-site, double blind placebo controlled study whose aim was to assess the efficacy of palatal implants as a treatment for mild to moderate OSA. Exclusion criteria were an AHI < 10 or > 40 based on the in-home baseline study, body mass index > 32 kg/m^2^, evidence of airway obstruction other than retro-palatal, prior airway surgery other than nasal, adenoid or tonsil, and presence of another sleep disorder (restless legs, insomnia, narcolepsy, etc.)

Thirty-seven patients from the placebo group underwent a baseline PSG and in-home sleep study followed by a second set of studies under the same conditions. The in-home studies were conducted approximately 60 days prior to the initial PSG studies (mean 59 ± 34 days). The time between the test and retest for PSG was almost five months (mean 140 + 35 days) and for the in-home studies was over six months (mean 188 + 47 days). The follow-up PSG and in-home studies were typically conducted within three weeks (mean 18 ± 21 days). Each subject had his or her test and retest PSG done at the same accredited sleep disorders center, with 12 subjects studied at the University of Cincinnati, 13 studied at the Medical College of Wisconsin, and 12 studied at Total Sleep Diagnostics of Indianapolis. The PSG data were acquired and scored by registered polysomnographic technicians applying the pre-2007 American Academy of Sleep Medicine guideline to classify apneas (i.e., 10-second cessation in airflow) and hypopneas (i.e., > 30% decrease in airflow and > 3% desaturation), and sleep staging (i.e., Rechtschaffen and Kales criteria) across sites. Total sleep times were 308 ± 61 SD minutes during the PSG test and 323 ± 50 SD minutes during the PSG retest.

The in-home studies were performed with Apnea Risk Evaluation System (ARES™) Unicorder (Advanced Brain Monitoring, Carlsbad, CA, USA). The ARES Unicorder measures oxygen saturation, pulse rate, airflow, respiratory effort, snoring levels, head movement, and head position from a wireless recorder self applied with a single strap to the forehead. Reflectance oximetry is used to obtain the SpO_2 _and pulse rate signals. Respiratory effort is derived from the measurement of changes in forehead venous pressure acquired using a combination of photoplethysmography and changes in surface pressure of the reflectance oximetry sensor, and head movement. Airflow is obtained via a nasal cannula and a pressure transducer. A calibrated acoustic microphone is used to acquire quantified snoring levels (dB). Accelerometers are used to measure head movement and derive head position. The recorder was designed to be easily affixed by the patient, and provide alerts during the study if poor quality airflow or SpO_2 _is detected so the device could be adjusted.

Automated scoring algorithms were applied off-line to detect sleep disordered breathing. The AHI was computed using a time-in-bed measure based on recording time with acceptable signal quality minus periods when the patient was upright or presumed to be awake based on actigraphy. Apneas, based on a 10-s cessation of airflow detected by the automated algorithms and hypopnea events that met the Medicare criteria required a 50% reduction and recovery in airflow, and a minimum 3.5% reduction in SpO_2 _and at least a 1.0% recovery were included in the apnea-hypopnea index (AHI-4%). Fifteen of the patients recorded two nights of in-home data during the test, two subjects completed a multi-night study during the retest. The total and valid recording times for the initial in-home test were 500 ± 211 and 451 ± 160 minutes respectively, and 406 ± 106 and 365 ± 104 minutes for the in-home retest.

Pearson correlations and Bland-Altman plots were used to assess the association in sleep disordered breathing between the test vs. retest data. Differences between the test and retest in demographic data and sleep parameters were tested with Student paired t-test.

## Results

**Figure 1:**
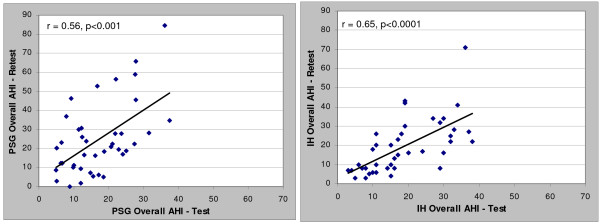
**Correlations of test-retest AHI values for: a) PSG and b) HST**.

**Figure 2:**
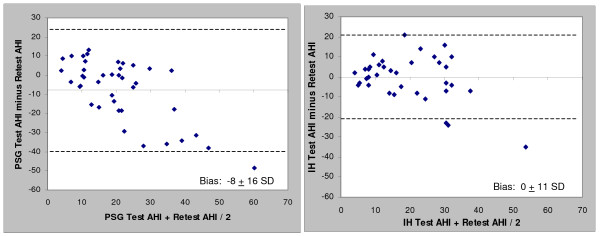
**Bland-Altman plots of test-retest AHI values for: a) PSG and b) HST**.

**Figure 3:**
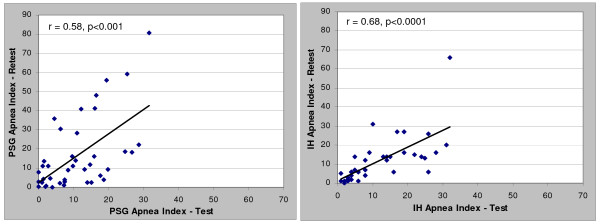
**Correlations of test-retest apnea index values for: a) PSG and b) HST**.

**Figure 4:**
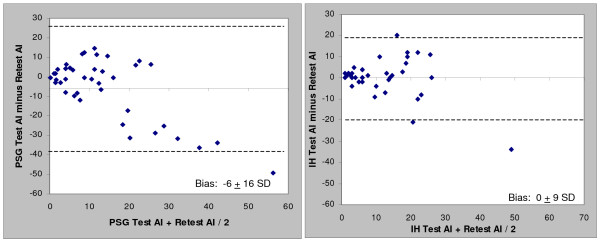
Bland-Altman plots of test-retest apnea index values for: a) PSG and b) HST

**Figure 5:**
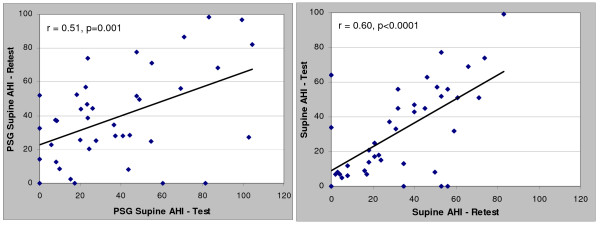
**Correlations of test-retest Supine AHI values for: a) PSG and b) HST**.

**Figure 6:**
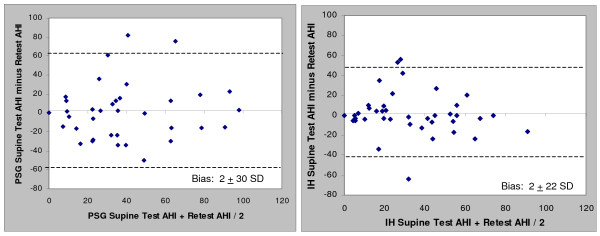
**Bland-Altman plots of test-retest Supine AHI values for: a) PSG and b) HST**.

**Figure 7:**
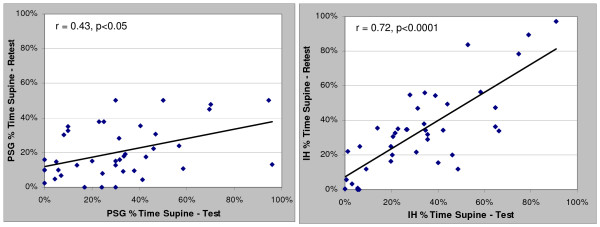
Correlations of test-retest percentage time Supine for: a) PSG and b) HST

Correlation and Bland-Altman plots between the test and retest data for the PSG and in-home studies (HST) are presented in Figures 1, 2, 3 and 4 for the overall AHI and apnea index. The in-home data consistently provided higher correlations and approximately 50% less variance than the PSG results. More importantly, the PSG data were biased toward higher AHI and apnea index values at retest while the in-home data were not. This trend toward reduced variability in the home recordings applied to the supine AHI data as well (Figures 5 and 6). The percentage of time supine was substantially more consistent when measured in the home (Figure 7). There was a bias toward increased time supine during the PSG-test vs. the PSG-retest (bias 12 ± 22%) while the in-home bias in the percentage time supine was neutral (bias 0 ± 16%). There were no changes in the subject's BMI and neck size between the test and retest. Bland-Altman plots revealed a slight bias toward a greater AHI values detected by the initial in-home sleep study as compared to the initial PSG study (Bias 1.8 ± 11 SD events/hour). When comparing the retest results, the PSG showing a bias toward greater AHI values vs. the in-home studies (Bias 5.6 ± 27 SD events/hour).

Table 1 shows the sample size required to obtain two standard levels of statistical power for treatment studies in which the desired outcome is a 50% reduction in AHI. Test-retest reliability is assumed at two levels: zero and the respective inter-class correlation values observed for each measure. The "No bias" rows consider that it is impossible to know the expected value of the change over time; it may be that it really is the case that subjects' conditions worsened as the PSG indicates and, hence the in-home results are positively biased for treatment effects (rather than the other way around). Thus these rows assume the statistical test would be purely a change score test against an unanchored, and not clinically meaningful, value. The observed bias rows incorporate the results presented in Figure 2 in which the PSG is biased in the opposite direction of a positive treatment effect. One would therefore need additional treatment effect to overcome the measurement bias to achieve an observed treatment effect that meets the gold standard of 50% reduction.

**Table 1:** Sample sizes required to provide nominal statistical power for 50% treatment effect within subject.

	ARES		PSG	
Assumption	1-β = .80	1-β = .90	1-β = .80	1-β = .90
No bias, no reliability	24	31	57	76
No bias, observed reliability	10	13	27	35
Observed bias, no reliability	26	34	3,499	4,684
Observed bias, observed reliability	11	14	1,541	2,062

Assumed α = .05 *Nearest whole number sample size to generate, at minimum, the nominal value.

## Discussion

This is the first study which compares AHI variability in untreated mild to moderate OSA patients studied with PSG and in-home with a substantial time between the test and retest (i.e., five to six months). When comparing the overall AHI and apnea index values, the in-home results provided approximately 50% less variability than the PSG data (Figures 2 and 4) and showed virtually no bias. Most of the PSG bias and variability came from the six subjects whose PSG-AHI had increased by more than 30 between the test and retest. By way of comparison, the ARES AHI increased by more than 20 in two of these six subjects, in one case it decreased by 21, and the change was less than 5 events/hr in the remaining three. If the six subjects (17%) are excluded from the analyses, the results from the two methods become quite similar (ARES AHI: bias = 0.6, SD = 8.5; PSG AHI: bias = -0.7, SD = 8.6). Neither the demographic data nor the sleep parameters (TST, % time supine) supported these six subjects being dropped as their results did not differ significantly with the rest of the cohort (all p > 0.1). At best, the variability might be explained by a first-night effect for PSG that is known to under-report the severity of the disease in OSA subjects [17-19].

The substantially lower apnea and hypopnea variability obtained with the in-home system was likely influenced by the use of the same equipment and auto-scoring algorithms. The in-home device and auto-scoring algorithms used in this study was described and validated in two previous studies. The first had 284 valid comparisons of the in-laboratory simultaneous PSG and ARES and 187 valid comparisons of the in-laboratory PSG with a separate two nights unattended self-applied ARES Unicorder [20]. The second study with 102 participants had 92 simultaneous in-laboratory comparisons and 86 in-home to in lab comparisons [21]. Both studies showed that the ARES had high sensitivity and specificity. It is uncertain whether the findings in this study apply to other in-home Level III sleep study devices.

A limitation of this study is that the conditions used to acquire the PSG data reflect the conventional practice at sleep centers, as opposed to employing extra-ordinary means to control variability. For example, it was previously reported that the depth of desaturation varies by equipment manufacturer [22] and the AHI used in this study was based on a 4% desaturation. However we could not ensure the same type of pulse-oximeter was used in all rooms and at all sites. Although the procedures used to score the sleep and respiratory events within and across the sleep centers were standardized, intra- and inter-rater reliability assessments were not made. We found no statistically significant differences in test-retest reliability among the three sleep centers. There was, however, a trend suggesting one facility provided more reliable results even though all three centers followed identical guidelines for data acquisition and scoring. This finding suggested that future multi-site PSG-based outcome studies should consider utilizing a single facility for the scoring of the data to improve test-retest reliability. Of interest, the overall AHI scored by multiple technicians in this study showed improved correlations as compared to a previous report in which a single technician scored all the records (r = 0.56 vs. 0.44) [19].

Another factor which may have impacted reliability for both the in-home and PSG studies was the selection of patients with AHI values between 10 and 40 based on the initial in-home study. Previous reports suggest that patients with mild to moderate OSA tend to have positional dependent severity that is more readily influenced by the time spent in the supine position.

Differences in the supine AHI variability were less severe with the ARES studies as compared to PSG may be explained by the percentage of time supine being more consistent in-home vs. in-lab. The correlations in the percent time supine for the in-home study in this group (r = 0.72) studied approximately five months apart was very similar to the results from a group studied under similar circumstances 40 days apart (r = 0.70) [18]. Patients spent less time supine in the lab during their retest, thus the expected impact of gravity on AHI does explain the bias toward increased PSG-AHI values upon retest. The in-home study recorded head position while the PSG studies used chest position, but it's uncertain what role this played in the supine positional results.

Several of the factors that might have impacted the test vs. retest AHI values were controlled by comparing laboratory and in-home results obtained under relatively similar circumstances. The subjects represented a homogenous group of subjects with baseline AHI values in the mild to moderate range, not excessively obese and without other sleep disorders. Although the initial in-home study was conducted two months earlier than the initial PSG study, the weight, BMI and neck circumference of the subjects did not significantly change between any of the in-home and PSG studies (all t-test p > 0.1). If a patient changed medication between the test and retest, it should have impacted both the PSG and in-home data similarly. In a previous study, Levendowski et al [19] was unable to relate differences in sleep architecture with differences in repeated measure PSG-AHI values. This study points toward a need for a well controlled prospective study to further clarify the issues which impact PSG variability.

The results from this study suggest major ramifications for the planning of future treatment studies. If one assumes no bias, and incorporates the observed reliability for the respective measures, the sample size required for PSG would be approximately three times that required for an ARES in-home study. Assuming the PSG is biased toward increasing values upon retest, the sample size required is more than 2 orders of magnitude larger than for the ARES and effectively impossible to implement. The bias toward increasing AHI values upon retest by PSG in this study (7.7 events/hour, p < .01) is very similar to the results reported by Levendowski et al. (i.e., 7 event/hour increase in PSG-AHI values at a 40 day retest) [19] and it is unlikely a coincidence. Other studies have also reported a "first-night" effect whereby PSG AHI values increase at retest [17,18].

## Conclusion

Test-retest variability in the sleep disordered breathing severity can be a confounding factor in assessing treatment outcomes. These data suggest that in-home studies provide a repeated measure of sleep disordered breathing less variable that that obtained by PSG at multiple sites. Guidelines developed to standardize the scoring of sleep and detection of related events (i.e., accreditation by the American Academy of Sleep Medicine) appears ineffective in controlling the inherent variability of OSA when measured by PSG. The acquisition of multi-night PSG studies is both difficult for subjects and very expensive. Investigators using PSG for assessing treatment outcomes should factor in the increased variability and tendency toward increased AHI values upon retest when selecting a sample size to ensure the study is adequately powered.

## Abbreviations

AHI: apnea/hypopnea index; PSG: polysomnography; OSA: obstructive sleep apnea; AI: apnea index; ARES: Apnea Risk Evaluation System

## Competing interests

DS and TW have no competing interest. DL, PW and DP are employees of Advanced Brain Monitoring. DL and PW are shareholders in Advanced Brain Monitoring, Inc.

## Authors' contributions

DL was the primary author and PW performed the primary editing. DS and TW managed the sites, which included recruitment, consent and acquisition of the PSG and in-home data. RO and DP conducted the statistical analyses presented in the paper.
